# Direct jet coaxial electrospinning of axon‐mimicking fibers for diffusion tensor imaging

**DOI:** 10.1002/pat.6073

**Published:** 2023-05-09

**Authors:** Chunyan Hu, Matthew Grech‐Sollars, Ben Statton, Zhanxiong Li, Fei Gao, Gareth R. Williams, Geoff J. M. Parker, Feng‐Lei Zhou

**Affiliations:** ^1^ College of Textiles and Clothing Qingdao University Qingdao China; ^2^ Department of Computer Science University College London London UK; ^3^ Lysholm Department of Neuroradiology, National Hospital for Neurology and Neurosurgery University College London Hospitals NHS Foundation Trust London UK; ^4^ Medical Research Council, London Institute of Medical Sciences Imperial College London London UK; ^5^ College of Textile and Clothing Engineering Soochow University Suzhou China; ^6^ Department of Radiology, Shandong Provincial Hospital, Cheeloo College of Medicine Shandong University Jinan China; ^7^ School of Pharmacy University College London London UK; ^8^ Centre for Medical Image Computing, Department of Medical Physics and Biomedical Engineering University College London London UK; ^9^ Bioxydyn Limited Manchester UK

**Keywords:** axon microstructure, coaxial electrospinning, diffusion phantoms, diffusion tensor imaging, hollow microfibers

## Abstract

Hollow polymer microfibers with variable microstructural and hydrophilic properties were proposed as building elements to create axon‐mimicking phantoms for validation of diffusion tensor imaging (DTI). The axon‐mimicking microfibers were fabricated in a mm‐thick 3D anisotropic fiber strip, by direct jet coaxial electrospinning of PCL/polysiloxane‐based surfactant (PSi) mixture as shell and polyethylene oxide (PEO) as core. Hydrophilic PCL‐PSi fiber strips were first obtained by carefully selecting appropriate solvents for the core and appropriate fiber collector rotating and transverse speeds. The porous cross‐section and anisotropic orientation of axon‐mimicking fibers were then quantitatively evaluated using two ImageJ plugins—nearest distance (ND) and directionality based on their scanning electron microscopy (SEM) images. Third, axon‐mimicking phantom was constructed from PCL‐PSi fiber strips with variable porous‐section and fiber orientation and tested on a 3T clinical MR scanner. The relationship between DTI measurements (mean diffusivity [MD] and fractional anisotropy [FA]) of phantom samples and their pore size and fiber orientation was investigated. Two key microstructural parameters of axon‐mimicking phantoms including normalized pore distance and dispersion of fiber orientation could well interpret the variations in DTI measurements. Two PCL‐PSi phantom samples made from different regions of the same fiber strips were found to have similar MD and FA values, indicating that the direct jet coaxial electrospun fiber strips had consistent microstructure. More importantly, the MD and FA values of the developed axon‐mimicking phantoms were mostly in the biologically relevant range.

## 1 INTRODUCTION

Axons in the central nerve system (CNS) are highly anisotropic fibrous structures with diameters of ~0.1–10 μm.^1^, ^2^, ^3^ Diffusion magnetic resonance imaging (dMRI) provides the ability to infer the microstructure and organization of tissues by monitoring the diffusion of water molecules within and between cells in vivo; changes in cellularity and fiber orientation affect water diffusion, and therefore macroscopically recorded diffusion MRI signals. There has been an increasing number of studies on probing changes in microstructure of the brain^4^ and spinal cord^5^ via diffusion MRI, not only as a diagnostic marker of diseases but also as a measure of treatment response to various therapies.

Multiple techniques^6^ and biophysical diffusion models^7^ of white matter are intensively exploited for assessing neurite fiber orientation using dMRI. Diffusion tensor imaging (DTI) and more sophisticated models of the dMRI signal can noninvasively reveal information of white matter microstructure within the spinal cord^5^ and brain.^8^ The majority of white matter models developed to date consist of two or three non‐exchanging compartments reflecting intra‐axonal restricted diffusion, extra‐axonal hindered diffusion and/or free diffusion of cerebrospinal fluid.^7^

As diffusion MRI becomes more sophisticated and microstructurally informative, it has become increasingly important to compare diffusion MRI estimates with reference measurements to assess the accuracy and precision of measurements. For example, a recent study compared fiber orientation dispersion in human brain white matter derived from dMRI data using a two‐compartment model with reference measures of dispersion acquired from polarized light imaging and histology of the tissue.^9^ However, histological material is limited as a reference, as changes in microstructure during fixation raise questions regarding the accuracy of any derived comparison, and the necessarily destructive nature of tissue sampling impedes routine use. Moreover, when wishing to compare the performance of dMRI microstructural measurements between scanning hardware or between centers, histological comparison is inherently of less use, as questions of relative scanner bias and precision come to the fore. It is in such settings that imaging phantoms can be of particular use. Imaging phantoms are a well‐characterized “gold standard” in terms of size and composition that can be used for evaluating MRI methods, assessing derived parameters and the limitations of diffusion models, in addition to providing an essential tool for comparing measurements between scanners.

Recent advances in MRI phantoms are summarized in Reference 10. The majority of microstructural phantoms currently in existence are used for the validation of imaging methods and/or microstructural models in brain dMRI. Among those microstructural phantoms, hollow poly(ɛ‐caprolactone) (PCL) microfibers generated by electrospinning^11^, ^12^, ^13^, ^14^ and hollow polypropylene (PP) fibers from melt‐spinning^15^, ^16^, ^17^ have shown a great potential to design and construct brain white matter‐mimicking MR phantoms, which can approximate both the micro‐scale cellular structure and the macro‐scale connections within the brain. Our previous studies on both 7T pre‐clinical and 3T clinical MR scanners have demonstrated both hydrophobic^11^, ^12^, ^13^ and hydrophilic^14^, ^18^ hollow PCL microfiber‐based phantoms can achieve excellent MR signal, MR measurement repeatability and chemical stability. In the case of hollow PP fiber phantoms, previous studies conducted on two 3T MRI systems^15^, ^16^ have provided evidence of their ability to validate algorithms for fiber tracking and the accuracy of diffusion MRI estimates of compartment size and packing density.

Despite efforts to promote uniformity and consistency in dMRI data collection, there remains the well‐recognized issue related to intra‐scanner^19^ and inter‐scanner^20^ variations in dMRI measurements. Ice‐water phantoms consisting of distilled water or sucrose solutions are widely used to assess multi‐system or multi‐center reproducibility of dMRI parameters in the brain.^21^, ^22^, ^23^ However, these phantoms are display isotropic non‐time‐dependent diffusion and hence they cannot validate dMRI estimates of anisotropy, of fiber orientation dispersion in white matter, or of microstructural properties. DTI phantoms containing melt‐spun hollow PP fibers have been employed to assess intra‐ and inter‐scanner variability in fractional anisotropy (FA) values using different imaging protocols on up to four 3T MR scanners, indicating substantial inter‐scanner differences.^24^, ^25^ The primary advantage of coaxial electrospun fiber phantoms over PP fiber phantoms lies in fiber size variation and fiber orientation control, which is expected to help generate dMRI data that more closely mimic those obtained from real biological tissue.

Coaxial electrospinning has become increasingly popular in creating core‐sheath structures for various functional applications.^26^, ^27^, ^28^, ^29^, ^30^, ^31^, ^32^ Hollow fibers, as a special core‐shell fibers with an empty core, are frequently generated using a combination of coaxial electrospinning and post‐treatment. However, the post‐treatment is not always required when appropriate shell and core polymer solutions are chosen, for example, PCL in CHCl_3_/DMF as shell and PEO/water as core.^33^ In the coaxial electrospinning process fibers can be collected in a range of forms from completely random, through uniaxially aligned orientation to patterned deposition by using an X‐Y translation stage‐based collector.^34^ This offers greater flexibility than melt‐spinning to prepare fiber phantoms with variable fiber orientation dispersion. For example, random and well‐aligned coaxial electrospun hollow fibers can be used to design more isotropic gray matter phantom.^35^

This study, the first‐of‐its‐kind, reports coaxial electrospun axon‐mimicking hydrophilic PCL‐PSi fibers phantom with variable fiber orientation and pore sizes and their use to characterize DTI measurements on a clinical MRI scanner. Coaxial electrospinning was first conducted using two combinations of shell and core solutions to generate hollow microfibers, followed by the microstructural characterization via scanning electron microscopy (SEM) to determine fiber orientation and porous structure. A phantom was then constructed from coaxial electrospun hollow hydrophilic PCL‐PSi microfibers. We investigated how the changes in fiber orientation dispersion and pore size in the phantom samples affect measurements of mean diffusivity (MD) and fractional anisotropy (FA) values of the phantom under room temperature and in ice‐water.

## 2 MATERIALS AND METHODS

### 2.1 Materials

PCL (number‐averaged molecular weight Mn = 80 kg mol^−1^), polyethylene oxide (PEO, viscosity‐average molecular weight Mv = 900 k) and the surfactant poly[dimethylsiloxane‐co‐[3‐(2‐(2‐hydroxyethoxy)ethoxy) propyl] methylsiloxane] (abbreviated as PSi) (Cat No. 480320) were obtained from Sigma‐Aldrich (Dorset, UK). The solvents chloroform (CHCl_3_) and N,N dimethyl‐formamide (DMF) were also purchased from Sigma Aldrich (Dorset, UK). Deionized water or chloroform was used to dissolve PEO.

### 2.2 Coaxial electrospinning of PCL and PCL‐PSi hollow microfibers

The experimental set‐up used in this study for preparing hollow microfibers was described previously.^34^ In brief, a coaxial spinneret, with two concentric needles, was filled with a shell solution of PCL or PCL‐PSi (outer needle, 16‐gauge [OD 1.65 mm, ID 1.19 mm]) and a core solution of PEO (inner needle, 22‐gauge [OD 0.72 mm, ID 0.41 mm]). The outer needle was connected to the positive electrode from a DC high voltage power supply (PS/FC30R04.0e22, Glassman High Voltage, UK), and the fiber collector, which was placed 5–15 cm below the tip of the concentric needles, was connected to the grounded electrode.

The shell/core solutions and process parameters for coaxial electrospinning are given in Table 1. These parameters guarantee a reasonably stable coaxial electrospinning process and were determined after a series of optimization experiments with respect to polymer concentrations, flow rates, and applied voltage. A wide rotating drum was used as a collector to align the fibers. The drum was positioned on an X‐Y translational stage and spun around X direction at controllable revolutions per minute (rpm), reciprocating along one track in the X direction at a tuneable speed from 1 mm/s, 4 mm/s to 8 mm/s, which allowed the fiber deposition to be spread uniformly over the collector. Once the solvents in outer and inner components in the deposited fibers evaporated, a solidified outer sheath was formed into hollow fibers. All experiments were conducted in a fume cupboard at ambient conditions. In a typical procedure for coaxial electrospinning, a solution of PCL‐PSi in CHCl_3_/DMF was used as the shell fluid and PEO in deionized water or CHCl_3_ acted as the core fluid. These two liquids were fed at a constant flow rate independently controlled by two syringe pumps.

**TABLE 1:** Summary of shell/core solutions and process parameters in coaxial electrospinning. (typical temperature of the environment is of 20–24°C, typical humidity is 28%–38%)

Set	Shell solution	Core solution	Process parameters (applied voltage/working distance/shell/core flow rate)
1	10 wt% PCL‐PSi (10/1 PCL/PSi, wt/wt) in CHCl_3_/DMF (8/2, wt/wt)	2.5 wt% PEO in CHCl_3_	9.0 kV/14.5 cm/3/0.35 mL/h 9.0 kV/14.5 cm/3/1.4 mL/h
2	10 wt% PCL‐PSi (10/1 PCL/PSi, wt/wt) in CHCl_3_/DMF (8/2, wt/wt)	4 wt% PEO in water	9.0 kV/5 cm/3/1.0 mL/h

### 2.3 Scanning electron microscopy

The detailed surface morphology and ultrastructure of the coaxial electrospun fibers was examined using a Philips XL30 FEG SEM or Quanta FEG 650 SEM operating at 5 kV under high vacuum. The coaxial electrospun fibers collected on the rotating drum were coated with thin gold/palladium (Au/Pd) alloy with a thickness of approximately 10 nm to increase their conductivity before SEM imaging. For imaging of fiber cross‐sections, fibers were cut using sharp scissors in liquid nitrogen. The pore size and porosity of coaxial electrospun fibers were measured using our previously reported methods.^36^

### 2.4 Analysis of fiber orientation and porous morphology

For the determination of the orientation of the coaxial electrospun fibers, the “Fourier Components” was performed using the “Directionality” plug‐in for Fiji ImageJ (http://fiji.sc/Fiji, Ashburn, VA).This calculates the spatial frequencies within an image over a set of radial directions. The Directionality method was used to generate normalized histograms revealing the amount of fibers present between 0° and 180°, from which the mean fiber orientation, fiber dispersion, and goodness of fit are extracted. Fibers oriented along the length of the strip were defined to be at a 90° orientation, while fibers oriented perpendicular to the length were at a 0° or 180° orientation.

ImageJ was used for automated analysis of pore‐size of the coaxial electrospun fiber strips, via the Analyze Particles build‐in plugin. The nearest distance (ND)^37^ plugin was used to estimate the distance between a pore and its *N* closest neighboring pores, which was taken as a parameter describing extra‐fiber pores in the cross‐section of the fiber strips. During the analysis, each pore was fitted with a circle with the center coordinates (X, Y), area *A*, diameter *D*_*I*_ and wall thickness *T*. The mean pore distance (*D*_*O*_) between the *i*th pore and its *N* closest pores is defined as

$$D_{\mathrm{Oi}}=\frac{1}{N}\sum_{1}^{N}\left(\sqrt{{\left(Y_{N}-Y_{i}\right)}^{2}+{\left(X_{N}-X_{i}\right)}^{2}}-\left(D_{\text{IN}}+D_{\mathrm{Ii}}\right)/2\right).$$

Based on Syurik's study,^38^ the coordination number *N* was set to be 3 in the present study. The mean pore distance (*D*_*O*_) of the *i*th pore was then normalized by its pore diameter (*D*_*Ii*_), expressed as

$$\delta =\frac{D_{\mathrm{Oi}}}{D_{\mathrm{Ii}}}.$$

In the case of hollow microfibers, the wall thickness contributes to the values of *D*_*O*_ and the value of *δ* since the Analyze Particles plugin measures the pore size based on the inner boundary of hollow microfibers. The circularity of the pores and pore size in all SEM images processed by ImageJ were set to be 0.5–1 and 10–500 μm^2^, unless otherwise stated, which should represent the majority of microstructural features expected in our samples.

### 2.5 Phantom design and construction

**FIGURE 1:**
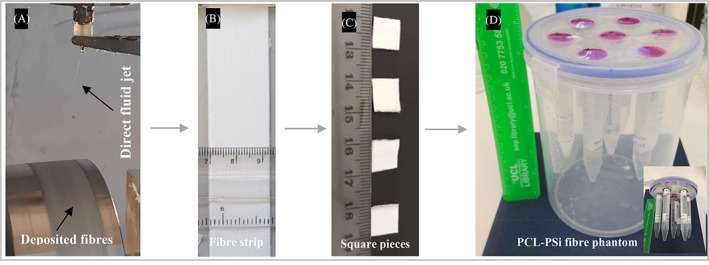
Schematic of direct jet coaxial electrospinning of hollow microfiber‐based diffusion tensor imaging phantoms. (A) A direct jet (only its top segment visible) and deposited fibers on a rotating drum mounted on an X‐Y translation stage (not shown). (B) Example fiber strip with cm scale, (C) Four 10 × 10 mm^2^ examples sectioned from the fiber strip with centimeter scale, (D) example hydrophilic PCL‐PSi fiber phantom comprising six samples (arranged along the perimeter) filled with water and 1 free water sample (in the center). Inset: seven phantom samples fixed on the cylinder container cap

The design and construction of phantoms is shown in Figure 1. A 6 h direct jet coaxial electrospinning process generated a fiber strip of ~0.5 mm thick and 345 mm long (Figure 1A,B). Around 20 layers of coaxial electrospun PCL‐PSi hollow fiber strips (~0.5 mm thick, 10 mm × 10 mm, Figure 1C) were packed into 15 mL plastic centrifuge tubes (17 mm outer diameter). The plastic tubes (up to 7 tubes) were then assembled on the custom‐made lid of a plastic cylinder (14 cm wide, 20 cm high; Figure 1D). In the case of PCL fiber phantoms, the fibers were aligned along the axis of the test tubes filled with cyclohexane; in the case of hydrophilic PCL‐PSi fiber phantoms, the fibers were aligned along the radial of plastic tubes filled with deionized water.

### 2.6 MRI acquisition

PCL‐PSi fiber phantoms testing on a Siemens Prisma 3T clinical MR scanner: hydrophilic phantoms were imaged once at standard room temperature (RT, 21.6°) and once in ice‐water. On the first scan, the phantom was filled with water and allowed to settle before being scanned. The phantom was then filled with ice‐water in a similar process as explained in a previous study,^21^ allowing each phantom to reach thermal equilibrium at about 0°C. Phantom temperatures were recorded at the end of each imaging session. The protocol included two repeat acquisitions of a standard PGSE‐DTI sequence: voxel size of 1.7 mm × 1.7 mm × 2 mm, field of view (FOV) of 220 mm x 220 mm, 30 gradient directions (*b* = 0, 1000 s mm^−2^), repetition time (TR) = 3200 ms, time to echo (TE) = 69 ms, acquisition time of 3 min 39 s, and using a 20‐channel head coil. DTI data were analyzed on the scanner by drawing regions of interest on one middle slice within each phantom to obtain the mean values and standard deviation for FA and MD.

### 2.7 Statistical analysis

The effect of the collector speed on fiber orientation and inner diameters of coaxial electrospun hollow fibers was assessed statistically by firstly determining the distribution of the data set using the Kolmogorov–Smirnov test.^39^ The data set was then assessed for homogenous variance using Levene's test. Data sets which fulfilled the Kolmogorov–Smirnov test and Levene's test criteria were assessed using ANOVA with a Tukey post‐test. Data sets which did not fulfill both test criteria were assessed using Kruskal–Wallis test (KW ANOVA) with Dunn's multiple comparisons post‐test or Mann–Whitney test. All statistical analyses were conducted at a 95% level of significance using OriginPro 2021 software (version 9.8.0.200 (Academic), OriginLab, Northampton, MA).

## 3 RESULTS AND DISCUSSION

### 3.1 Coaxial electrospinning of PCL‐PSi fiber strips using PEO/chloroform as core solution

Based on the previously successful formation of hollow fiber structure in narrow PCL‐PSi fiber bundles,^14^ two PCL‐PSi fiber strips were collected after slightly modifying the experimental parameters. The strips were produced using core flow rates of 0.35 and 1.4 mL/h, while other parameters were kept constant in the coaxial electrospinning of 10 wt% PCL‐PSi/CHCl_3_ + DMF shell solution and 2.5 wt% PEO/CHCl_3_ core solution. The strips were denoted PCL‐PSi‐0.35 and PCL‐PSi‐1.4. The solution of 4 wt% was found to be too viscous to spin.

**FIGURE 2:**
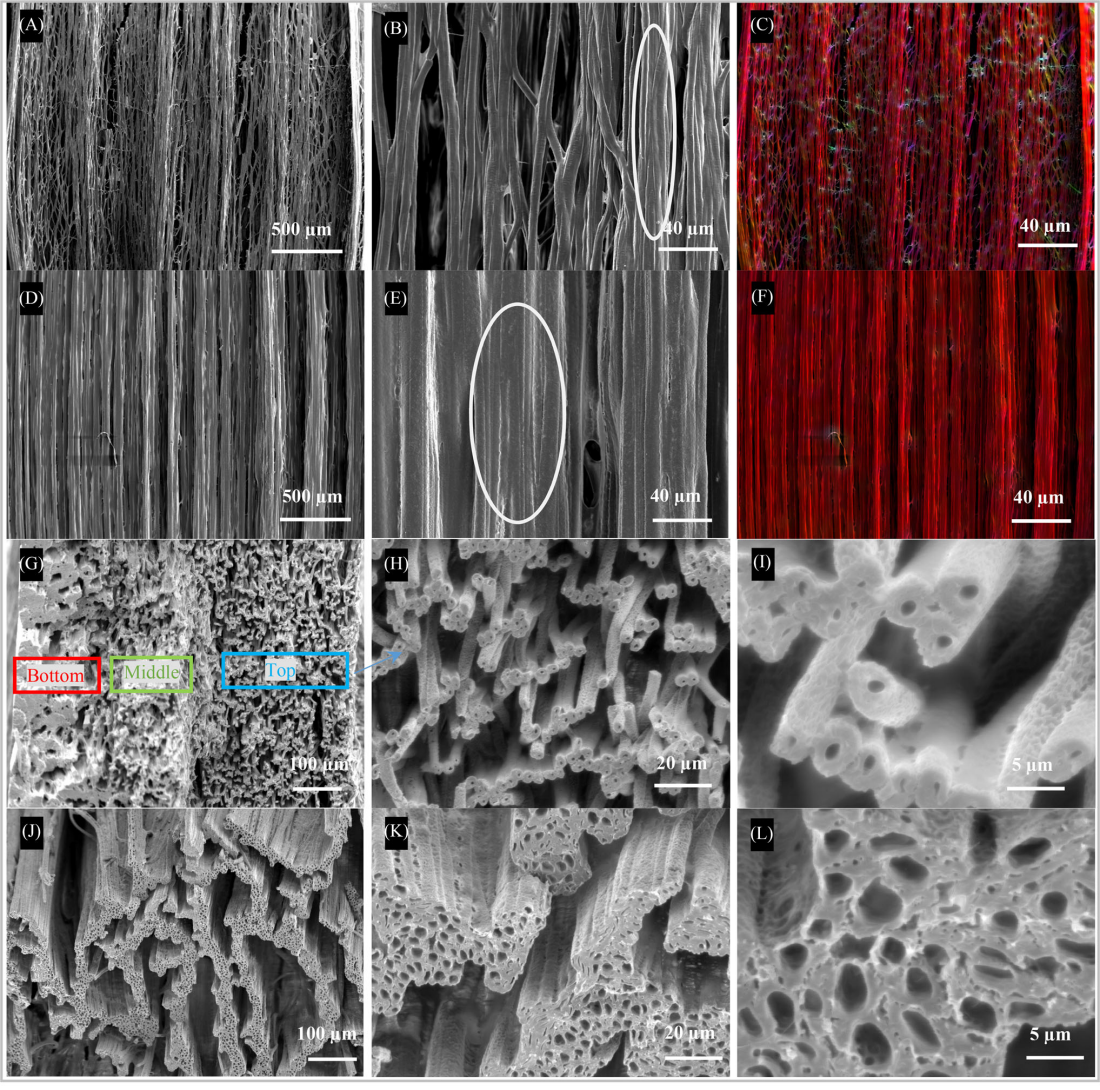
Scanning electron microscopy micrographs of top surface and cross‐section of direct jet coaxial electrospun PCL‐PSi fiber strips using 0.35 and 1.4 mL/h core flow rates. Fiber orientations in (A, B) PCL‐PSi‐0.35 and (D, E) PCL‐PSi‐1.4 fiber strips. (C, F) Color coded images for visual representation of fiber orientation in (A) and (D). Cross‐sections of the PCL‐PSi‐0.35 mL/h (G–I) and PCL‐PSi‐1.4 mL/h strips (J–L). Other parameter settings: shell solution—10% PCL‐PSi/CHCl_3_ + DMF, core solution—2.5 wt% PEO/CHCl_3_, applied voltage—9.0 kV, working distance—14.5 cm, shell flow rate—3 mL/h, operation time—6 h, X‐Y translation stage speed—1 mm/s, rotating collector speed—800 rpm

As shown in Figure 2, the fibers are well aligned (Figure 2A,B,D,E) in each PCL‐PSi fiber strip. As reflected in the color coded images (Figure 2C,F), the dominant orientation angle of the fibers was calculated to be 87.8° and 89.9°, respectively. The compound jet in these two coaxial electrospinning processes was straight and was therefore referred to as direct jet coaxial electrospinning, which had a lower downward axial velocity than the jet composed of straight and bending segments in conventional electrospinning. The surface velocity of the rotating collector reached ~9.2 m/s when rotating at 800 rpm, aligning and importantly stretching the deposited fibers along the circumferential direction of the collector. Some fibers in the PCL‐PSi‐0.35 mL/h strip partially merged with their neighbors (highlighted region by the ellipse in Figure 2B); in contrast, the fibers in the PSi‐1.4 strip are found to merge with their neighbors far more uniformly (see the ellipse in Figure 2E), but did not form a continuous film.

As shown in Figure 2G–L, the cross‐sections of the fibers were apparently different between the PCL‐PSi‐0.35 and PCL‐PSi‐1.4 strips. Each strip had a distinct cross‐sectional structure (as shown in Figure 2G,J). In the PCL‐PSi‐0.35 strip, across its whole thickness there were three regions with gradually changing porous structures, as highlighted in three colored rectangular boxes in Figure 2G. In the PCL‐PSi‐1.4 strip, several separated layers comprising 3–5 individual hollow fibers were observed and each fiber layer had a thickness of a few tens of microns (as indicated Figure 2J). The fibers in the bottom region of the 0.35 mL/h strip (the side attached to the collector surface) almost completely merged to a depth of ~160 μm (as indicated by red box), but the fibers became gradually separated and hollow (Figure 2H) from the ~160 μm thick middle region (as indicated by green box) to the ~250 μm thick top region (as indicated by yellow box). The fibers in the top region were partially merged with neighboring fibers (Figure 2I), which is consistent with the observation of their surface morphology (Figure 2B). For the 1.4 mL/h strip, fiber merging, as shown by cross‐sectional SEM (Figure 2K), was also similar to that shown by its surface topography (Figure 2E). Besides the microstructural differences, the sizes of hollow fibers at 0.35 mL/h were much smaller than those of in the PCL‐PSi‐1.4, as shown in Figure 2I,L, The area‐weighted inner diameter^36^ of the hollow fibers in the two strips were 0.9 ± 0.08 μm (only in the top fibrous region) and 2.4 ± 0.02 μm (fibers across different layers), respectively. The individual fibers in the PCL‐PSi‐0.35 strip also had much higher ratios of wall thickness to pore size than those in the PCL‐PSi‐1.4 strip and in fiber strips produced using a PEO/water core solution, which contributed to the higher normalized pore distance *δ*, as shown in Figure 1.

**FIGURE 3:**
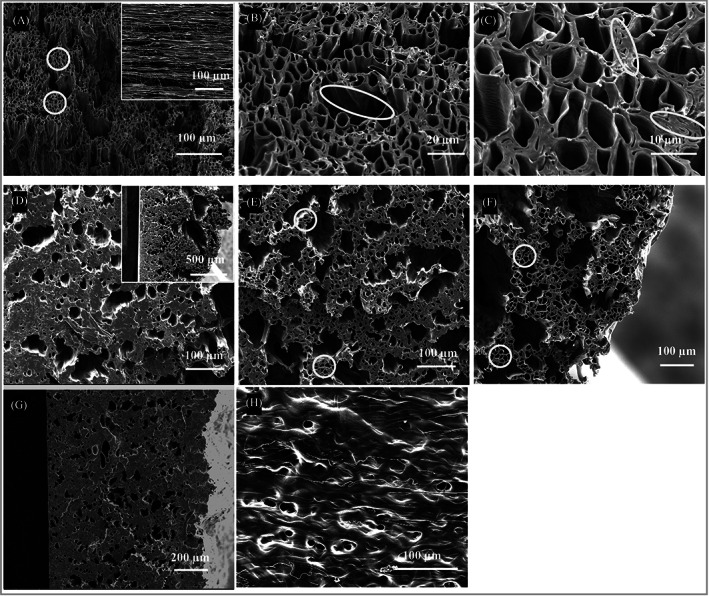
Coaxial electrospun PCL‐PSi fibers from 10% PCL‐PSi/CHCl_3_ + DMF shell solution with 4 wt% PEO/water core solution using 3.0/1.0 mL/h shell/core flow rate, 9.0 kv, 5 cm, 6 h production time, 800 rpm at translation speed of (A–C) 1 mm/s, (D–F) 4 mm/s (top, middle and bottom region), (G, H) 8 mm/s

Based on the experimental results, it was evident that both the PCL‐PSi‐0.35 and PCL‐PSi‐1.4 fiber strips are not as close to the axon microstructure as fibers (e.g., Figure 3A–C) generated via coaxial electrospinning using PEO/water as the core solution. Varying the core and/or shell solutions in coaxial electrospinning could effectively result in hollow microfibers in a relatively short‐time period as previously reported.^40^ On the other hand, the variations of the core and/or the shell solution compositions, which resulted in a slowdown or acceleration of the evaporation rate, can dramatically affect the final form of hollow microfibers in their bulk structure, such as in the case of fiber strips. The potential effect on the hollow microfibers of CHCl_3_ in the core solution is discussed below in comparison with the use of water as the core solvent.

### 3.2 Coaxial electrospinning of PCL‐PSi fibers using PEO/water as core solution

**FIGURE 4:**
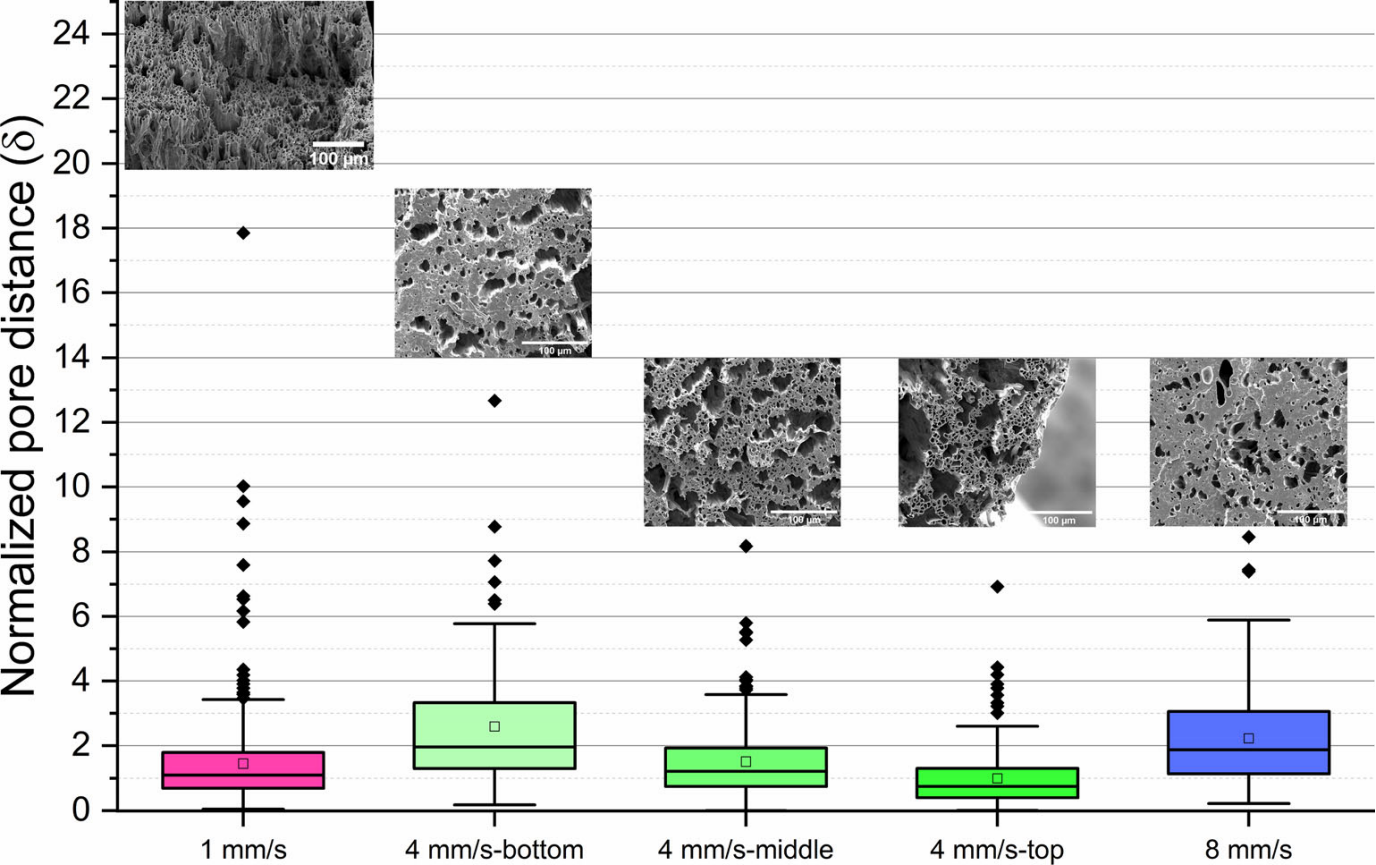
Boxplots of the dispersions of normalized pore distance of three coaxial electrospun PCL‐PSi fiber strips from 10% PCL‐PSi/CHCl_3_ + DMF shell solution with 4 wt% PEO/water core solution using 3.0/1.0 mL/h shell/core flow rate, 9.0 kv, 5 cm, 6 h production time, 800 rpm at translation speed of 1, 4, and 8 mm/s

When 4 wt% PEO/water was used as the core solution, three PCL‐PSi fiber strips were collected using different X‐Y translation speeds, namely 1, 4, and 8 mm/s. The resultant PCL‐PSi fibers have dramatically different cross‐sections, as shown in Figure 4. Among these three samples, the fibers in the PCL‐PSi‐1 strip are hollow across the whole thickness (Figure 3A) but merge with their neighbors, resulting in some extra‐fiber void space. Figure 3B shows that these void spaces (as highlighted by an ellipse) often have irregular shapes and much larger size than the inner diameters of the hollow fibers. In addition to the dominant hollow microstructure, some hollow fibers are found to be collapsed, leading to closely packed regions, as highlighted by ellipses in Figure 3C. The typical extra‐fiber void space between partially merged fibers, with in the 1 mm/s sample, is also revealed by the longitudinal surface in the inset SEM in Figure 3A.

When the translation speed was increased to 4 mm/s, the cross‐section of the PCL‐PSi‐4 strip was found to vary from highly merged to porous structure from the bottom to the top of the strip (Figure 3D–F), which is very similar to the microstructural change in the PCL‐PSi‐0.35 fiber strip (Figure 2G). At the bottom region of the PCL‐PSi‐4 fiber strip, hollow fibers are barely observed due to the complete fusion into a region of 250 μm thickness, with an increasing number of irregular pores (Figure 3D) in the upper region. Hollow, but still significantly merged, fibers are present in the middle region of fiber strip (Figure 3E). As shown in Figure 3F, the hollow fibers become much less fused in the top layer of the strip, where some significant delamination occurs (as shown by the inset in Figure 3D). Those fibers in the PCL‐PSi‐8 sample become completely fused as no discrete fibers are observed across the whole thickness (Figure 3G), which almost resembles the microstructure of the bottom region in the PCL‐PSi‐4 fiber strip. The fiber fusion microstructure is also shown by the longitudinal image of the strip (Figure 3H).

Figure 4 shows the dispersions of normalized pore distance (*δ*) of the three fiber strips with changing translation speed. For the PCL‐PSi‐4 fiber strips, three dispersions of *δ* are illustrated here due to variable porous structures across the cross‐section. Their dispersions of normalized pore distance reveal that the fibers in three strips are either partially packed with pore size larger than wall thickness or highly merged. There are some outliers of *δ* (e.g., up to 18 in PCL‐PSi‐1 strip), corresponding to relatively large void space and/or merged areas present in these fiber strips.

The normalized pore distance of the PCL‐PSi‐1 fiber strip has considerable overlaps with the middle and top regions of PCL‐PSi‐4 strip, indicating there are lots of small local areas that have similar porous microstructure, with highly packed hollow fibers, as shown in circled area in Figure 3A,E,F. In the PCL‐PSi‐4 fiber strip, the normalized pore distance exhibits gradually smaller and narrower from the strip bottom to top region, which is not seen in PCL‐PSi‐1 and PCL‐PSi‐8 fiber strips. For the nonoverlapping region, the values of normalized pore distance (*δ*) in the middle are higher and in the top regions are lower, revealing that there are some more merged regions as shown Figure 3E and some thinner walls between fibers in Figure 3F. The distribution of normalized pore distance of the PCL‐PSi‐8 fiber strip is similar to the bottom region of the PCL‐PSi‐4 fiber strip (*p* = 0.58, KW ANOVA).

In the coaxial electrospinning process, a core‐shell fluid jet is ejected from a spinneret and deposited on a grounded electrode with a 15–20 cm gap in less than 10 ms.^41^ The rapid solvent evaporation, which is even quicker (i.e., in a few milliseconds^42^), occurs in two main stages, one from the shell and the other from the core solution. The initial stage of the evaporation process results in the formation of core‐shell structured fibers filled with solvent and the final stage of evaporation of the core solvent through a solidified shell mainly determines the final form of the tubular fibers: they can adopt collapsed, partially collapsed or non‐collapsed (cylindrical) shapes.^43^ In particular, the final stage of evaporation heavily relies on the diffusion coefficient of the core solvent through the fiber shell.^43^ An increasing water content in the mixed solvent system water/EtOH changed the hollow PCL microfiber from tubular (6/4, wt/wt) to partially (7/3, 8/2, wt/wt) or totally collapsed structure (1/0, wt/wt) in coaxial electrospinning using a working distance between the spinneret and the collector of 16 and 20 cm.^43^

The above solvent diffusion‐based mechanism behind the hollow microfiber formation in coaxial electrospinning cannot fully explain some experimental observations here. For example, in the coaxial electrospinning process using PEO/water as core solution, hollow PCL‐PSi fibers were still formed (Figure 3A–C), though in our previous study with a 23 or 15 cm working distance (close to that used in Reference 43), completely or partially collapsed PCL fibers were also formed.^14^ Here, the formation of hollow fiber structure could be attributed to the relatively shorter working distance (5 cm) where the jet comprises only a straight segment. The straight jet could undergo the final stage of evaporation on the rotating collector on which it is subject to much higher mechanical stretching than the jet in the longer working distances. Based on the solvent diffusion mechanism in Reference 43, the jet deformation, that is, thinning, results in a decrease in the internal volume of the tubular fibers formed on the first stage and thus the decrease in the pressure difference inside and outside the tubular fibers caused by solvent evaporation, which prevents the fiber collapsing or buckling to some extent. It is also worth noting that hollow PCL‐PSi fibers were formed from a PEO/CHCl_3_ core solvent in the previous section but had different pore geometries from those fibers generated from PEO/water or PEO/water + EtOH core solution, even though the working distance used was similar (e.g., 16–20 cm in Reference 33). The formation of this unique porous structure on fibers could be explained by the dual effect of CHCl_3_ on fibers formation including high diffusion coefficient and good solvent to the shell polymer while the solvent–water or ethanol exerts only the diffusion effect.

The effect of varying the X‐Y translation speed between 1 and 8 mm/s on PCL‐PSi fiber cross‐sections is consistent with previous finding for the coaxial electrospinning PCL fiber strips created with X‐Y translation speeds between 0.2 and 5 mm/s.^36^ In the present coaxial electrospinning of hollow PCL‐PSi fibers using 5 cm working distance, the landing speed of the straight jet should be much higher than that (ca. 15–20 mm/s) in near‐field electrospinning^44^ but much lower than that in conventional electrospinning (ca. 0.7–1.8 m/s).^45^ That is to say, the landing speed of the PCL‐PSi fibers is far more than the translation speed of the collector, resulting in the formation of multiple fibers along the jet direction before fibers spread on the collector surface. As previously explained, the wet fiber strands deposited early would have less time to dry before a new fiber layer are deposited on the rotating collector when moving at higher translation speeds, leading to fiber fusion in the PCL‐PSi fiber strips.

The above analysis suggests that the rotating collector relevant parameters (rotation speed and translation speed), together with the proposed final‐stage solvent evaporation should be taken in account in the coaxial electrospinning process of hollow microfibers, in particular, bulk structures.

### 3.3 Co‐ES of hollow PCL‐PSi microfibers with variable collector speed

**FIGURE 5:**
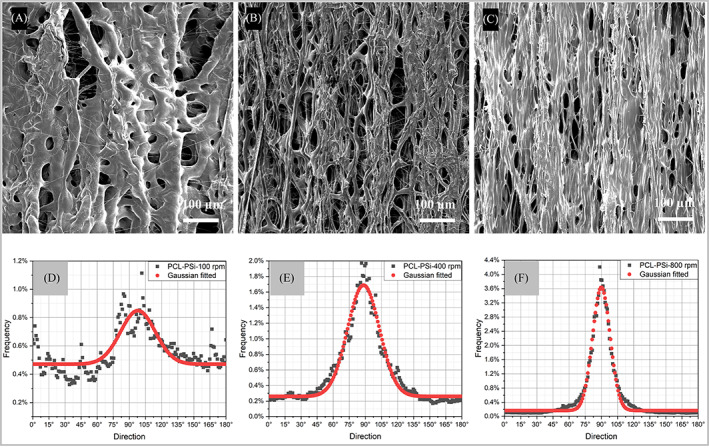
Scanning electron microscopy micrographs of longitudinal sections of direct jet coaxial electrospun PCL‐PSi fiber strips using various rotating speeds and their orientation distributions. (A, D) 100 rpm; (B, E) 400 rpm; (C, F) 800 rpm. Coaxial electrospinning process parameters: 10 wt% PCL‐PSi/CHCl_3_ + DMF shell and 4 wt% PEO/water core using 3.0/1.0 mL/h shell/core flow rate, 1.8 kV/cm electric field strength, and a 5 cm working distance

**FIGURE 6:**
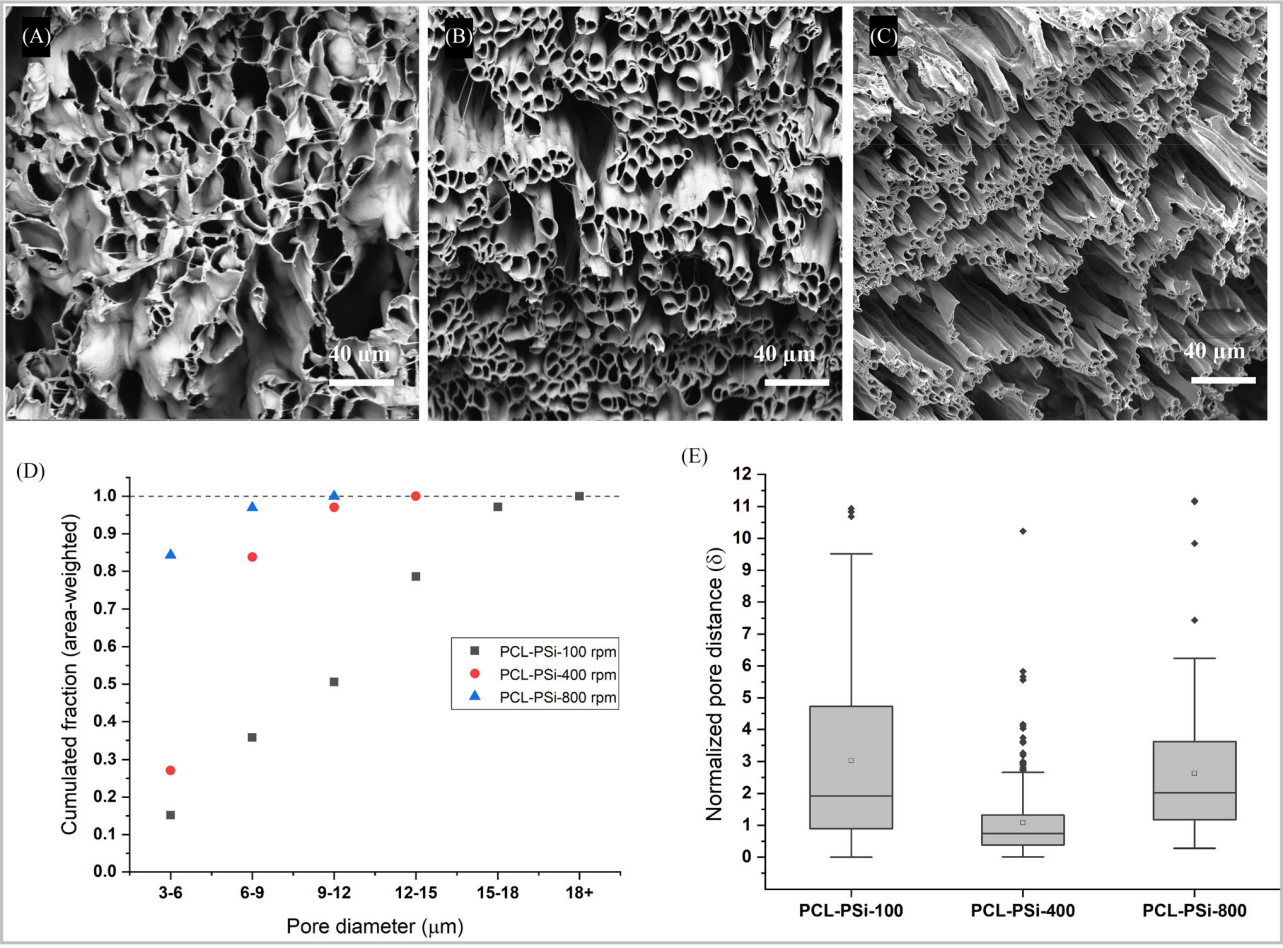
Scanning electron microscopy images of cross‐sections of coaxial electrospun PCL‐PSi fibers from 10 wt% PCL‐PSi/CHCl_3_ + DMF shell and 4 wt% PEO/water core using 3.0/1.0 mL/h shell/core flow rate, 1.8 kV/cm electric field strength, and a 5 cm working distance at three rotating speeds of (A) 100 rpm; (B) 400 rpm; (C) 800 rpm. (D) Histograms of area‐weighted inner diameter distributions; (E) boxplots of normalized pore distance and dispersion of fiber orientation. Scale bars in (A–C): 40 μm

Similar to PCL fiber strips, three PCL‐PSi fiber strips were prepared using similar process settings, denoted as PCL‐PSi‐100, 4000, and 800, respectively. Representative longitudinal and cross‐sectional morphologies from each PCL‐PSi fiber strip are shown in Figures 5 and 6. In general, the longitudinal surface morphology of PCL‐PSi strips as shown in Figure 5A–C appears to replicate that observed in PCL fiber strips (Figure 5A–C). Some merging with neighboring fibers can be seen in the three PCL‐PSi strips, resulting in lateral connection between fibers via ribbon‐like fibers and void space between those merged fibers. However, the fibrous structures are still dominant, though significantly merged fibers locally formed some small area of non‐fibrous film. Table 2 summarizes the mean fiber orientation and orientation dispersion in SEM images shown in Figure 5A–C via Directionality. The fibers in three PCL‐PSi fiber strips were also well aligned, as indicated by the mean fiber orientation values at around 90°. Figure 5D–F shows the histograms of the fiber orientation dispersions via Directionality, which were significantly different (*p* << 0.05, KW ANOVA).

**TABLE 2:** Dominant fiber direction, pore sizes and diffusion tensor imaging measurements of co‐ES hollow PCL‐PSi hollow microfiber phantoms

Collector speed			PCL‐PSi fiber phantoms		
Orientation, pore size, MR measurement			100 rpm	400 rpm	800 rpm
Mean fiber orientation ± STDEV (fiber percentage in the range) by Directionality			98.0° ± 16.2° (0.45)	88.3° ± 14.9° (0.65)	90.0° ± 8.2°(0.73)
Number‐averaged median pore size ± IQR (μm)			6.3 ± 4.7	6.2 ± 2.2	4.6 ± 1.1
Normalized pore distance (*δ*) Median ± IQR (μm)			1.9 ± 3.8	0.7 ± 0.9	2.0 ± 2.4
Area‐weighted pore size (*ɛ*) ± standard deviation (μm)			10.6 ± 2.0	7.3 ± 0.4	5.1 ± 0.2
Mean diffusivity (MD) (μm^2^/ms)	RT (21.6°C)	Repeat 1	1.03 ± 0.10	0.88 ± 0.12	1.05 ± 0.07
			1.04 ± 0.04	0.93 ± 0.17	1.08 ± 0.87
	Ice‐water (0.16°C)	Repeat 2	0.66 ± 0.08	0.52 ± 0.05	0.65 ± 0.06
			0.66 ± 0.03	0.55 ± 0.07	0.65 ± 0.04
Fractional anisotropy (FA)	RT (21.6°C)	Repeat 1	0.24 ± 0.05	0.61 ± 0.04	0.59 ± 0.05
			0.22 ± 0.02	0.55 ± 0.07	0.53 ± 0.05
	Ice‐water (0.16°C)	Repeat 1	0.19 ± 0.06	0.51 ± 0.08	0.45 ± 0.05
			0.17 ± 0.02	0.46 ± 0.09	0.43 ± 0.04

The PCL‐PSi fiber strips also had similar final thickness (~0.5 mm) to the PCL fiber strips above. As shown in Figure 6A–C, hollow fibers and relatively large void extra‐fiber space were the main features of the cross‐sections of the three PCL‐PSi strips and became obviously thinner with increasing rotation speed. The area‐weighted pore diameter distributions of hollow microfibers in the three strips were presented in Figure 6D. The pore diameter range of the PCL‐PSi‐100 strip spanned 3 μm‐18+ μm diameters, which was wider than the PCL‐PSi‐400 and PCL‐PSi‐800 strips with ranges of 3–15 μm and 3–12 μm, respectively. There are dramatic changes in area‐weighted inner diameter from 10.6 ± 2.0 μm of PCL‐PSi‐100, through 7.3 ± 0.4 μm of PCL‐PSi‐400 to 5.1 ± 0.2 μm of PCL‐PSi‐100. As shown by Figure 6E, the distribution of normalized pore distance of the PCL‐PSi‐800 fiber strip entirely overlapped with that of PCL‐PSi‐100 fiber strip (*p* = 1, KW ANOVA). However, a significantly narrower dispersion of normalized pore distance was seen in PCL‐400 fiber strip (*p* << 0.05, KW ANOVA).

The normalized pore distance and dispersion of fiber orientation were also compared between PCL fiber and PCL‐PSi fibers. In terms of the dispersion of normalized pore distance, there was no dramatic difference between the PCL‐100 and PCL‐PSi‐100 strips (*p* = 0.18, Mann–Whitney). The differences between the PCL‐400 and PCL‐PSi‐400 strips and between the PCL‐800 and PCL‐PSi‐800 strips were significant, (*p* << 0.05, Mann–Whitney). In terms of the dispersion of fiber orientation in the two types of fiber strips, the difference between the PCL‐100 and PCL‐PSi‐100 strips was not significant (*p* = 0.39, Mann–Whitney), but there were significant differences between the PCL‐400 and PCL‐PSI‐400 strips and between the PCL‐800 and PCL‐PSi‐800 strips (*p* << 0.05, Mann–Whitney). These results indicated that the addition of PSi in the PCL shell could affect the porous microstructure and fiber orientation only at relatively high collector speeds (400 and 800 rpm). However, it should be mentioned that the value of normalized pore distance is prone to be influenced by the SEM sample preparation in which freeze facture could deform the pores especially in large size hollow fibers shown in both Figure 6A–C. Therefore, here the apparent effect of PSi on the porous structure in two types of strips is not conclusive.

### 3.4 Hydrophilic PCL‐PSi phantom testing on a 3T clinical MR scanner

MR images were acquired following the scan of the six PCL‐PSi fiber phantom samples using a 3T clinical MRI scanner. MD and FA values of the six PCL‐PSi fiber phantoms were measured. For the PCL‐PSi‐100, −400 and −800 fiber phantoms, the MD and FA values for the two repeat scans, acquired at 21.6°C and in ice‐water are listed in Table 2. Both MD and FA values for the two repeats of each of the three phantoms were less than 6% of mean value for MD, and less than 11% of mean value for FA (a maximum difference of 0.05 μm^2^/ms for MD and 0.06 for FA), indicating high consistent quality across each fiber strip. For the PCL‐PSi‐800 fiber phantom sample, its MD value was 14.3%–22.8% higher than previously reported MD (0.81–0.90 μm^2^/ms) and its FA value is 20.3%–24.4% lower than the FA value (0.74–0.78) of three PCL‐PSi‐800 fiber samples reported in Reference 40. It has to be noted that the MD and FA values in Reference 40 were the median values acquired on the same model of MRI scanner in another imaging center (3T Siemens Prisma at UCL) and temperature was not reported, which might contribute to the discrepancies in MD and FA values.

There was a decrease in MD of 14.5% from the PCL‐PSi‐100 to PCL‐PSi‐400, and then an increase by 19.3% to its original value in the PCL‐PSi‐800 fiber phantom, while the area‐weight pore sizes of hollow fibers decreased by 54.5%. As discussed in Section 3.4, there was a considerable decrease in the distribution of normalized pore distance between the PCL‐PSi‐100 and PCL‐PSi‐400, and then a considerable increase in the distribution between PCL‐PSi‐400 and PCL‐PSi‐800, but there was not a significant difference between the PCL‐PSi 100 and PCL‐PSi‐800 strips. These variations in the dispersion of the normalized pore distance could explain the fact that there was no clear trend in the MD values of the three PCL‐PSi fiber phantom samples. There was a sharp increase in FA value of 154% from the PCL‐PSi‐100 to PCL‐PSi‐400, followed a slight decrease by 3.3% to PCL‐PSi 800. The significant difference in the dispersion of fiber orientation between PCL‐PSi‐100 and PCL‐PSi‐400 fiber strips should be responsible for the sharp increase in FA values of the corresponding phantoms; the relatively larger standard deviation in fiber orientation in PCL‐PSi‐400 phantom than PCL‐PSi‐800 one could explain the slight change in FA value.

The advantage of using ice‐water is that the temperature was fixed and not affected by the temperature inside the scanner room. The MD and FA values acquired from PCL‐PSi fiber phantoms in ice‐water at 0.16° were shown in Table 2. As expected, MD and FA values were lower than those acquired at room temperature due to the slower diffusion of water molecules at lower temperatures. Also, the trends of MD and FA values in ice‐water were found to be consistent with the those acquired at room temperature for three phantom samples.

From the above analysis, one can conclude that the dominant microstructural parameters defining MR measurements in fiber phantoms are the normalized pore distance and the dispersion of fiber orientation. These two parameters can more closely reflect the ground truth microstructure of coaxial electrospun strips, and therefore more appropriately explain the changes in MD and FA values.

## 4 CONCLUSION

In conclusion, we have demonstrated that the direct jet coaxial electrospinning technique permits the formation of 3D macrostructures—fiber strips of axon‐mimicking hollow microfibers with variable microstructure, that is, pore distance, pore size, and fiber orientation. Two spinnable polymers (PEO and PCL) were used as the core and shell components, and by selecting an appropriate solvent in the core and specific collector rotating speed and translation speed, one could obtain hydrophilic PCL‐PSi fiber strips with highly porous cross‐section and anisotropic fiber orientation. The constructed axon‐mimicking diffusion phantoms based on PCL‐PSi fiber strips allowed us to perform diffusion MR scans on a clinical MR scanner. The MD and FA values of axon phantoms were largely determined by the pore size, pore distance and fiber orientation. We found that the normalized pore distance and fiber orientation dispersion of developed phantoms could be used to interpret the variations in their DTI measurements. Similar to our previously demonstrated quality of PCL fiber strips,^12^, ^13^ the microstructural quality of PCL‐PSi fiber strips was also consistent. More importantly, the MD and FA values of these developed axon phantoms were mostly in the range of or comparable to those reported for previously developed phantoms, and therefore are biologically relevant.

## Data Availability

The data that support the findings of this study are available on request from the corresponding author. The data are not publicly available due to privacy or ethical restrictions.

## References

[1] Liewald D , Miller R , Logothetis N , Wagner HJ , Schüz A . Distribution of axon diameters in cortical white matter: an electron‐microscopic study on three human brains and a macaque. Biol Cybern. 2014;108(5):541‐557. 25142940 10.1007/s00422-014-0626-2 PMC4228120

[2] Perge JA , Niven JE , Mugnaini E , Balasubramanian V , Sterling P . Why Do Axons Differ in Caliber? J Neurosci. 2012;32(2):626‐638. 22238098 10.1523/JNEUROSCI.4254-11.2012 PMC3571697

[3] Duval T , Saliani A , Nami H , et al. Axons morphometry in the human spinal cord. Neuroimage. 2019;185:119‐128. 30326296 10.1016/j.neuroimage.2018.10.033

[4] Alexander DC , Dyrby TB , Nilsson M , Zhang H . Imaging brain microstructure with diffusion MRI: practicality and applications. NMR Biomed. 2019;32(4):e3841. 29193413 10.1002/nbm.3841

[5] Zaninovich OA , Avila MJ, Kay M, Becker JL, Hurlbert RJ, Martirosyan NL. The role of diffusion tensor imaging in the diagnosis, prognosis, and assessment of recovery and treatment of spinal cord injury: a systematic review. Neurosurg Focus. 2019;46(3):E7. 10.3171/2019.1.FOCUS18591 30835681

[6] Tournier JD . Diffusion MRI in the brain—theory and concepts. Prog Nucl Magn Reson Spectrosc. 2019;112‐113:1‐16. 10.1016/j.pnmrs.2019.03.001 31481155

[7] Jelescu IO , Budde MD . Design and validation of diffusion MRI models of white matter. Front Phys Ther. 2017;5(61):16. 10.3389/fphy.2017.00061 PMC5947881 29755979

[8] Viallon M , Cuvinciuc V , Delattre B , et al. State‐of‐the‐art MRI techniques in neuroradiology: principles, pitfalls, and clinical applications. Neuroradiology. 2015;57(5):441‐467. 25859832 10.1007/s00234-015-1500-1

[9] Mollink J , Kleinnijenhuis M , Cappellen van Walsum AM , et al. Evaluating fibre orientation dispersion in white matter: comparison of diffusion MRI, histology and polarized light imaging. Neuroimage. 2017;157:561‐574. 28602815 10.1016/j.neuroimage.2017.06.001 PMC5607356

[10] Drobnjak I , Neher P , Poupon C , Sarwar T . Physical and digital phantoms for validating tractography and assessing artifacts. Neuroimage. 2021;245:118704. 34748954 10.1016/j.neuroimage.2021.118704

[11] Zhou F‐L , Hubbard PL , Eichhorn SJ , Parker GJM . Coaxially electrospun axon‐mimicking fibers for diffusion magnetic resonance imaging. ACS Appl Mater Interfaces. 2012;4(11):6311‐6316. 23135104 10.1021/am301919s

[12] Hubbard PL , Zhou FL , Eichhorn SJ , Parker GJM . Biomimetic phantom for the validation of diffusion magnetic resonance imaging. Magn Reson Med. 2015;73(1):299‐305. 24469863 10.1002/mrm.25107

[13] Grech‐Sollars M , Zhou FL , Waldman AD , Parker GJM , Hubbard Cristinacce PL . Stability and reproducibility of co‐electrospun brain‐mimicking phantoms for quality assurance of diffusion MRI sequences. Neuroimage. 2018;181:395‐402. 29936312 10.1016/j.neuroimage.2018.06.059

[14] Zhou F‐L , Li Z , Gough JE , Hubbard Cristinacce PL , Parker GJM . Axon mimicking hydrophilic hollow polycaprolactone microfibres for diffusion magnetic resonance imaging. Mater Des. 2018;137(Supplement C):394‐403. 29307950 10.1016/j.matdes.2017.10.047 PMC5727678

[15] Guise C , Fernandes MM , Nóbrega JM , Pathak S , Schneider W , Fangueiro R . Hollow polypropylene yarns as a biomimetic brain phantom for the validation of high‐definition fiber tractography imaging. ACS Appl Mater Interfaces. 2016;8(44):29960‐29967. 27723307 10.1021/acsami.6b09809

[16] Fan Q , Nummenmaa A , Wichtmann B , et al. Validation of diffusion MRI estimates of compartment size and volume fraction in a biomimetic brain phantom using a human MRI scanner with 300 mT/m maximum gradient strength. Neuroimage. 2018;182:469‐478. 29337276 10.1016/j.neuroimage.2018.01.004 PMC6043413

[17] Fan Q , Nummenmaa A , Wichtmann B , et al. A comprehensive diffusion MRI dataset acquired on the MGH Connectome scanner in a biomimetic brain phantom. Data Brief. 2018;18:334‐339. 29896520 10.1016/j.dib.2018.03.021 PMC5996223

[18] Amy McDowell MGH , Zhou F , Feiweier T , Parker GJM , Clark CA . *Time dependence and stability of diffusion tensor metrics in a hydrophilic, electrospun, water‐perfused, hollow fiber phantom at 3T* in *ISMRM, Abstract No. 3556*. 2019.

[19] Bisdas S , Bohning DE , Bešenski N , Nicholas JS , Rumboldt Z . Reproducibility, interrater agreement, and age‐related changes of fractional anisotropy measures at 3T in healthy subjects: effect of the applied b‐value. Am J Neuroradiol. 2008;29(6):1128‐1133. 18372415 10.3174/ajnr.A1044 PMC8118811

[20] Teipel SJ , Reuter S , Stieltjes B , et al. Multicenter stability of diffusion tensor imaging measures: a European clinical and physical phantom study. Psychiatry Res: Neuroimaging. 2011;194(3):363‐371. 10.1016/j.pscychresns.2011.05.012 22078796

[21] Grech‐Sollars M , Hales PW , Miyazaki K , et al. Multi‐centre reproducibility of diffusion MRI parameters for clinical sequences in the brain. NMR Biomed. 2015;28(4):468‐485. 25802212 10.1002/nbm.3269 PMC4403968

[22] Malyarenko D , Galbán CJ , Londy FJ , et al. Multi‐system repeatability and reproducibility of apparent diffusion coefficient measurement using an ice‐water phantom. J Magn Reson Imaging: JMRI. 2013;37(5):1238‐1246. 23023785 10.1002/jmri.23825 PMC3548033

[23] Moreau B , Iannessi A , Hoog C , Beaumont H . How reliable are ADC measurements? A phantom and clinical study of cervical lymph nodes. Eur Radiol. 2018;28(8):3362‐3371. 29476218 10.1007/s00330-017-5265-2 PMC6028847

[24] Wilde EA , Provenzale JM , Taylor BA , et al. Assessment of quantitative magnetic resonance imaging metrics in the brain through the use of a novel phantom. Brain Inj. 2018;32(10):1265‐1275. 10.1080/02699052.2018.1494855 30169993

[25] Provenzale JM , Taylor BA , Wilde EA , Boss M , Schneider W . Analysis of variability of fractional anisotropy values at 3T using a novel diffusion tensor imaging phantom. Neuroradiol J. 2018;31(6):581‐586. 30037296 10.1177/1971400918789383 PMC6243462

[26] Yu P , Zhang J , Long J . Coaxial mechano‐electrospinning of oriented fibers with core‐shell structure for tactile sensing. Polym Adv Technol. 2023;34(3):821‐831.

[27] Ge R , Ji Y, Ding Y, Huang C, He H, Yu D‐G. Electrospun self‐emulsifying core‐shell nanofibers for effective delivery of paclitaxel. Front Bioeng Biotechnol. 2023;11:1112338. 36741747 10.3389/fbioe.2023.1112338 PMC9892910

[28] Bahmani E , Dizaji BF , Talaei S , et al. Fabrication of poly(ϵ‐caprolactone)/paclitaxel (core)/chitosan/zein/multi‐walled carbon nanotubes/doxorubicin (shell) nanofibers against MCF‐7 breast cancer. Polym Adv Technol. 2023;34(2):789‐799.

[29] Zhang Z , Yang K , Han X , Yu X , Cheng Z . Novel mosquito repellent fiber mat containing nepeta essential oil prepared by coaxial electrospinning. Polym Adv Technol. 2022;33(9):2943‐2951.

[30] Dai T , Qin Z , Wang S , et al. A novel nanofibrous film with antibacterial, antioxidant, and thermoregulatory functions fabricated by coaxial electrospinning. Polym Adv Technol. 2022;33(12):4062‐4071.

[31] Huang X , Jiang W , Zhou J , Yu DG , Liu H . The applications of ferulic‐acid‐loaded fibrous films for fruit preservation. Polymers. 2022;14(22):4947. 36433073 10.3390/polym14224947 PMC9693208

[32] Han D , Steckl AJ . Coaxial electrospinning formation of complex polymer fibers and their applications. ChemPlusChem. 2019;84(10):1453‐1497. 31943926 10.1002/cplu.201900281

[33] Dror Y , Salalha W , Avrahami R , et al. One‐step production of polymeric microtubes by co‐electrospinning. Small. 2007;3(6):1064‐1073. 17315262 10.1002/smll.200600536

[34] Zhou F‐L , Hubbard PL , Eichhorn SJ , Parker GJM . Jet deposition in near‐field electrospinning of patterned polycaprolactone and sugar‐polycaprolactone core–shell fibres. Polymer. 2011;52(16):3603‐3610.

[35] Ye AQ , Hubbard Cristinacce PL, Zhou F‐L, Yin Z, Parker GJM, Magin RL. *Diffusion tensor MRI phantom exhibits anomalous diffusion*. 2014 36th Annual International Conference of the IEEE Engineering in Medicine and Biology Society; 2014. 10.1109/EMBC.2014.6943698 PMC4605561 25570066

[36] Zhou F‐L , Parker GJM , Eichhorn SJ , Hubbard Cristinacce PL . Production and cross‐sectional characterization of aligned co‐electrospun hollow microfibrous bulk assemblies. Mater Charact. 2015;109:25‐35. 26702249 10.1016/j.matchar.2015.09.010 PMC4659418

[37] Haeri MaH M . ImageJ plugin for analysis of porous scaffolds used in tissue engineering. J Open Res Softw. 2015;3(1):e1.

[38] Syurik J , Schwaiger R , Sudera P , et al. Bio‐inspired micro‐to‐nanoporous polymers with tunable stiffness. Beilstein J Nanotechnol. 2017;8:906‐914. 28503401 10.3762/bjnano.8.92 PMC5405679

[39] Musciacchio L , Mardirossian M , Guagnini B , et al. Rifampicin‐loaded electrospun polycaprolactone membranes: characterization of stability, antibacterial effects and urotheliocytes proliferation. Mater Des. 2022;224:111286.

[40] Fenglei Zhou AM , Seunarine K , Hall MG , et al. *Axon‐mimicking hydrophilic fibre phantoms for diffusion MRI*. ISMRM, Abstract No.3636; 2019.

[41] Reneker DH , Yarin AL, Zussman E, Xu H. Electrospinning of nanofibers from polymer solutions and melts. In: Aref H , van der Giessen E , eds. Advances in Applied Mechanics. Elsevier; 2007;41:43‐195, 345‐346.

[42] Reddy CS , Arinstein A , Avrahami R , Zussman E . Fabrication of thermoset polymer nanofibers by co‐electrospinning of uniform core‐shell structures. J Mater Chem. 2009;19(39):7198‐7201.

[43] Arinstein A , Avrahami R , Zussman E . Buckling behaviour of electrospun microtubes: a simple theoretical model and experimental observations. J Phys D Appl Phys. 2009;42(1):015507.

[44] Sun D , Chang C , Li S , Lin L . Near‐field electrospinning. Nano Lett. 2006;6(4):839‐842. 16608294 10.1021/nl0602701

[45] Reneker DH , Kataphinan W , Theron A , Zussman E , Yarin AL . Nanofiber garlands of polycaprolactone by electrospinning. Polymer. 2002;43(25):6785‐6794.

